# Bilateral Choanal Atresia in a 65-Year-Old Female: A Case Report and Literature Review

**DOI:** 10.1155/crot/5584900

**Published:** 2025-11-05

**Authors:** Maraam Al Qout, Abdullah Alkarni, Abdulaziz Alaraifi, Mohammad Almahdi

**Affiliations:** ^1^Department of Otolaryngology-Head and Neck Surgery, Aseer Central Hospital, Abha, Saudi Arabia; ^2^Division of Otolaryngology-Head and Neck Surgery, Department of Surgery, King Abdulaziz Medical City, Ministry of National Guard Health Affairs, Riyadh, Saudi Arabia; ^3^King Abdullah International Medical Research Center, Riyadh, Saudi Arabia

**Keywords:** adult, bilateral choanal atresia, nasal obstruction, transnasal repair

## Abstract

**Background:**

Choanal atresia (CA) is a congenital condition characterized by occlusion of the nasal airway due to failure of recanalization during embryological development. It is more commonly unilateral and typically presents during infancy. In contrast, bilateral CA is a neonatal emergency that often manifests as respiratory distress at birth. The presentation of bilateral CA in late adulthood is extremely rare.

**Case Description:**

This case presents an unusual case of an elderly patient diagnosed with bilateral CA at 65 years. She presented with a primary complaint of lifelong bilateral nasal obstruction and denied any symptoms suggestive of chronic rhinosinusitis. Examination revealed bilateral choanal obstruction with no visible openings in the nasal cavity. The patient underwent endoscopic transseptal repair of bilateral CA, which included perforation of the atretic plate, posterior septectomy, and flap reconstruction. Postoperative evaluation demonstrated bilaterally patent choanae, and the patient remained asymptomatic, with no further complications.

**Conclusion:**

This case highlights a rare presentation of bilateral CA diagnosed in late adulthood. Although typically detected in the neonatal period, bilateral CA can occasionally remain undiagnosed for decades. This patient represents the oldest reported case of bilateral CA in the literature, emphasizing the variability in clinical presentation and the potential for delayed diagnosis.

## 1. Introduction

Choanal atresia (CA) is a congenital malformation of the nasal airway characterized by an occlusion between the nasal cavity and nasopharynx. This results from the failure of recanalization of the nasobuccal membrane during the seventh week of embryonic development [1]. It occurs more frequently in females than in males, with a ratio of 2:1, and has an estimated incidence of 1 in 8000 live births [2]. Unilateral CA is more common, accounting for approximately 60% of the cases. It often remains asymptomatic in neonates and rarely causes severe respiratory distress, sometimes presenting later in adulthood [1]. In contrast, bilateral CA is a neonatal emergency, typically manifesting as episodes of cyanosis and apnea shortly after birth [3]. Herein, we present a case of a 65-year-old woman diagnosed with bilateral CA, a condition typically detected at birth.

## 2. Case Presentation

**Figure 1:**
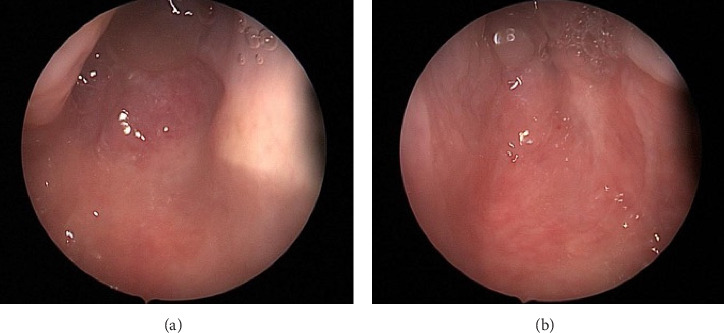
(a) Rigid endoscopic view showing complete obstruction of the right choana. (b) Rigid endoscopic view showing complete obstruction of the left choana.

**Figure 2:**
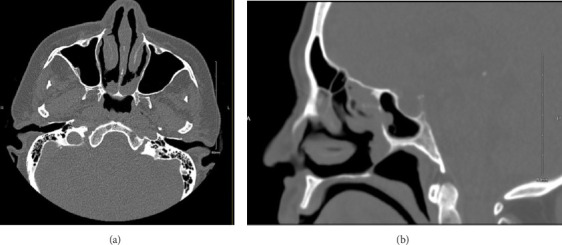
(a) Axial noncontrast CT scan of the paranasal sinuses (bone window) showing bilateral choanal atresia. (b) Sagittal noncontrast CT scan of the paranasal sinuses (bone window) showing choanal atresia.

A 65-year-old Saudi woman with a history of hypertension, diabetes mellitus, and bronchial asthma presented to the clinic with a complaint of lifelong bilateral nasal obstruction. She described the obstruction as persistent and associated with anosmia and intermittent mucoid nasal discharge. She denied any history suggestive of chronic rhinosinusitis, including postnasal drip, facial pain or pressure, and frontal headaches. She never sought medical management or intervention for her condition. Transnasal 0-degree endoscopic evaluation revealed bilateral complete absence of the choanal openings, which were surrounded by polypoidal tissue in proximity to the choanae (Figure 1). No other significant anatomical abnormalities were observed in the nasal cavities. A computerized tomography (CT) scan of the paranasal sinuses revealed bilateral CA with minimal mucosal thickening of the maxillary sinuses and mild opacification of the ethmoid air cells and sphenoid sinuses (Figure 2). These findings further confirmed the diagnosis and provided additional details on the sinonasal anatomy prior to potential surgical intervention. Regarding her comorbidities, the patient was managed by a family physician for hypertension, diabetes mellitus, and asthma, all of which were well controlled with medications. Prior to the operation, she was evaluated in the anesthesiology clinic for preoperative clearance and was classified as ASA class III.

**Figure 3:**
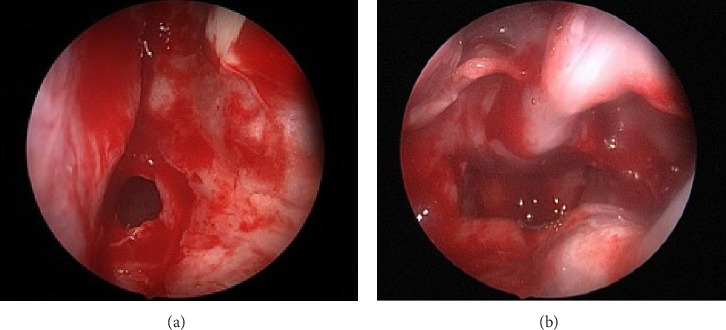
(a) Rigid endoscopic view showing the first puncture after raising the flap. (b) Rigid endoscope through the right side showing the common cavity post transseptal repair.

**Figure 4:**
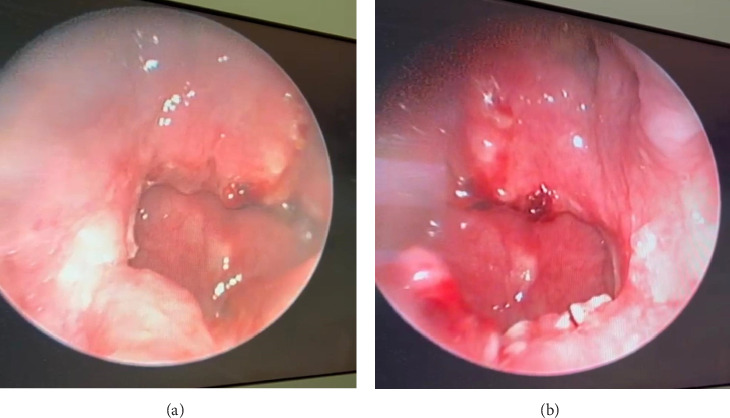
Rigid endoscopic views from the right (a) and left (b) sides at 6-week follow-up show well-healed, bilateral patent choanae following transseptal repair, with no evidence of stenosis.

The patient underwent endoscopic transnasal surgical repair of bilateral CA under general anesthesia. A 4-mm, 0-degree Hopkins telescope was used to examine the bilateral nasal cavities, revealing mixed bony and membranous CA with a thick vomer bone. Lidocaine (1%) with epinephrine (1:100,000) was injected into the posterior nasal septum, followed by a right-sided Killian incision and elevation of right mucoperiosteal flap. The atretic portion of the right nasal cavity was perforated transseptally using a suction freer, creating an initial passage to the postnasal space. The opening was then enlarged using a microdebrider, followed by posterior septectomy and removal of the bony junction between the atretic plate and vomer while preserving the mucoperiosteal flap bilaterally. The redundant mucosa was excised, and a common postnasal cavity was created, fully exposing the nasopharynx and the bilateral torus tubarius. Subsequently, the right and left mucoperiosteal flaps were predesigned to cover the postnasal common cavity along the roof and floor, respectively. A reassessment confirmed that the flaps were well positioned and a wide postnasal cavity had been achieved (Figure 3). Hemostasis was achieved using neuropatties soaked in xylometazoline and suction electrocautery. Finally, bilateral silastic breathable sheets were applied and secured in place using 2-0 Prolene sutures. The patient tolerated the procedure well, and no complications were observed. During postoperative follow-up visits at 2 weeks and 3 months, endoscopic examination of the nasal cavities revealed bilateral patent choanae with no stenosis (Figure 4).

## 3. Discussion

While unilateral CA is often asymptomatic and may go undiagnosed until later in life, bilateral CA typically presents as a neonatal emergency due to severe respiratory distress [1]. However, rare cases of bilateral CA diagnosed in adulthood have been reported [4, 5].

In 1755, Roederer reported the first instance of bilateral CA [6]. Several theories have been proposed to explain its embryological development, including the persistence of the buccopharyngeal membrane, Hochstetter's nasobuccal membrane, abnormal mesodermal tissue within the nasal choana, and misdirection of mesodermal flow due to local factors [7]. The condition is life-threatening in neonates, presenting with cyanosis, apnea, and respiratory distress, as newborns are obligate nasal breathers [1, 8, 9]. Therefore, the occurrence of undiagnosed bilateral CA persisting into adulthood is exceptionally rare. Carpenter and Neel (1977) studied 36 patients with unilateral or bilateral CA. Among them, twelve had bilateral CA, with eight diagnosed before 10 months of age, one at 16 months, one at 2 years, one at 15 years, and only one at 33 years [10]. These findings reinforce that bilateral CA is not typically considered a preliminary diagnosis for bilateral nasal obstruction in adults.

A review of the literature revealed 17 reported cases of bilateral CA in adults. As shown in Table 1, most patients were diagnosed in the second or third decade of life, whereas the present case involves a patient in her sixth decade. For instance, Aksoy et al. reported a 23-year-old patient with bilateral CA who presented with bilateral nasal obstruction and nasal discharge since birth [15]. The majority of the reported cases were females, including the current patient. Most cases had no associated abnormalities or disorders, while four cases were associated with telecanthus, hypogammaglobulinemia, pycnodysostosis, and Tessier number 3 facial cleft.

The current patient had hypertension, diabetes mellitus, and bronchial asthma; however, she had no associated congenital anomalies or syndromic disorders. Similar to this case, the most common presenting symptoms in the literature were nasal obstruction, rhinorrhea, and anosmia. Among the 17 reported cases, only two patients had a history of prior surgical intervention. The first underwent multiple procedures for cleft lip, palate, and nasal deformities, which may have contributed to the development of CA [9]. The second patient had surgical repair for bilateral CA as a newborn but later experienced recurrence [23]. Overall, only two patients were reported to have recurrence following surgical intervention. Importantly, all patients, including the present case, achieved favorable postoperative outcomes, with patent choanae documented during follow-up visits.

Our case represents the oldest documented instance in the literature to date. This patient experienced lifelong nasal obstruction without significant respiratory distress during infancy or childhood, suggesting possible partial obstruction or adaptation to oral breathing patterns. The latter is most likely because the examination showed no openings in the choana. This aligns with the theory proposed by Miller et al., who posited that infants are not strictly obligate nasal breathers and can adapt to oral breathing in the presence of nasal obstruction [10].

The management of bilateral CA in adults typically involves surgical intervention to establish nasal airway patency. The transnasal endoscopic approach is favored because of its effectiveness and minimally invasive nature. In this case, the patient underwent successful endoscopic transnasal repair, resulting in patent choanae and resolution of symptoms. This outcome is consistent with other reports advocating endoscopic techniques as a reliable treatment modality [13–16].

## 4. Conclusion

Bilateral CA is a rare congenital condition typically diagnosed in the neonatal period due to respiratory distress. However, in exceptional cases, it can remain undiagnosed until adulthood, as seen in this 65-year-old patient. This case adds to the growing evidence supporting the need to consider CA in adults presenting with lifelong nasal obstruction, even in the absence of significant respiratory symptoms. It also highlights the remarkable adaptability of some individuals to congenital nasal obstruction through compensatory mouth breathing. The successful surgical repair using an endoscopic transnasal approach further reinforces the efficacy of this technique in restoring nasal patency and improving quality of life.

## Figures and Tables

**Table 1:** Summary of reported adult cases of bilateral choanal atresia.

Author	Year	Age and gender	Associated abnormalities	Primary complaint	History of trauma or surgery or radiation	Reoccurrence	Surgical approach	Adjuvant therapies (stenting and mitomycin)	Outcome and follow-up	Reference
Carpenter et al.	1977	33	None	Not mentioned	Not mentioned	Not mentioned	Intranasal perforation	Not mentioned	Not mentioned	[8]
Latifi et al.	1999	22 Female	None	Bilateral nasal obstruction Anosmia	None	None	Transnasal endoscopic	None	Patent	[11]
Panda et al.	2004	22 Male	Telecanthus	Bilateral nasal obstruction Mucoid rhinorrhea Anosmia	None	None	Transnasal endoscopic	ET size 6 placed for 6 weeks	Patent	[12]
El-Sawy et al.	2006	24 Female	Hypogammaglobulinaemia	Bilateral nasal obstruction Anosmia	None	Yes	Transnasal endoscopic	None	Patent	[13]
Yasar et al.	2007	51 Female	None	Bilateral nasal obstruction Rhinorrhea Episodic respiratory distress	None	None	Transnasal endoscopic	ET size 7.5 placed for 7 days	Patent	[14]
Aksoy et al.	2009	23 Female	None	Bilateral nasal obstruction Rhinorrhea	None	None	Transnasal endoscopic	Topical mitomycin-C applied	Patent	[15]
Tinoco et al.	2010	34 Female	None	Bilateral nasal obstruction Rhinorrhea	None	None	Transnasal endoscopic	None	Patent	[3]
Chaudhary et al.	2010	Female	None	Bilateral pansinusitis	None	None	Transnasal endoscopic	None	Not mentioned	[16]
Tatar et al.	2012	53 Female	None	Difficulty of nasal breathing	None	None	Transnasal endoscopic	None	Patent	[17]
Bakir et al.	2014	21 Female	None	Bilateral nasal congestion Rhinorrhea	None	None	Transnasal endoscopic	NC size 18 placed for 3 weeks	Patent	[18]
Verma et al.	2016	20 Female	None	Nasal obstruction Rhinorrhea Snoring Anosmia	None	None	Transnasal endoscopic	None	Patent	[2]
Anajar et al.	2017	18 Male	None	Nasal obstruction Rhinorrhea Snoring Anosmia	None	None	Transnasal endoscopic	None	Patent	[19]
Durmaz et al.	2017	23 Female	Pycnodysostosis	Nasal congestion	None	None	Transnasal endoscopic	None	Patent	[20]
Sung et al.	2020	19 Female	Tessier number 3 facial cleft	Nasal obstruction rhinorrhea	Yes	None	Transnasal endoscopic	Foley catheter size 18-Fr placed for 8 weeks	Patent	[9]
Mengi et al.	2020	60 Female	None	Nasal congestion Rhinorrhea Snoring Anosmia	None	None	Transnasal endoscopic	ET size 6 placed for 3 weeks	Patent	[21]
Sutikno et al.	2021	27 Female	None	Bilateral nasal obstruction Snoring Anosmia	No	None	Transnasal endoscopic	None	Patent	[22]
Vieira et al.	2024	42 Female	None	Nasal obstruction Rhinorrhea Anosmia	Yes	Yes	Transnasal endoscopic	None	Patent	[23]
This case	2025	65 Female	None	Bilateral nasal obstruction Rhinorrhea	None	None	Transnasal endoscopic	None	Patent	This case

Abbreviations: ET, endotracheal tube; NC, Nelaton catheter.
